# Survival of U.S. Military Service Members with Hodgkin Lymphoma by Post-9/11 Deployment Status

**DOI:** 10.1158/2767-9764.CRC-26-0220

**Published:** 2026-08-21

**Authors:** Steven Gibson, Sarah Darmon, Wendy Cozen, Helen Ma, Matthew Rendo, Joshua L. Fenderson, James Aden, Michael Morris, Christin B. DeStefano

**Affiliations:** 1 Hematology/Oncology Service, Department of Medicine, Brooke Army Medical Center, JBSA Fort Sam Houston, Fort Sam Houston, Texas.; 2 Henry M. Jackson Foundation, Rockville, Maryland.; 3 Department of Medicine, School of Medicine, University of California, Irvine, Irvine, California.; 4 VA Long Beach Healthcare System, Long Beach, California.; 5 Wright-Patterson Air Force Base, Dayton, Ohio.; 6 Graduate Medical Education, Brooke Army Medical Center, JBSA Fort Sam Houston, Fort Sam Houston, Texas.; 7 Pulmonary/Critical Care Service, Department of Medicine, Brooke Army Medical Center, JBSA Fort Sam Houston, Fort Sam Houston, Texas.; 8 Walter Reed National Military Medical Center, Bethesda, Maryland.

## Abstract

Classic Hodgkin lymphoma (CHL) commonly affects adolescents and young adults (AYA), including service members (SM). The impact of deployment on CHL outcomes remains unknown. TRICARE beneficiaries diagnosed with CHL from 2001 to 2022 were identified through the Department of War cancer registry. Deployment history came from the Defense Manpower Data Center, and mortality data came from the National Death Index. Mortality was compared between SMs with and without post-9/11 deployment, and associations with deployment-specific variables were evaluated using Cox models adjusted for demographic and clinical factors. An exploratory analysis was performed to assess the survival of AYA SMs by race and to compare mortality with the Surveillance, Epidemiology, and End Results (SEER) database. Among 752 SMs identified, 369 (49%) had deployed after 9/11. Deployment was not associated with overall mortality [adjusted hazard ratio (aHR), 1.64; 95% confidence interval (CI), 0.84–3.19] or disease-specific mortality (aHR, 0.67; 95% CI, 0.13–3.55). No deployment-specific variable meaningfully influenced mortality. There were 21 out of 417 (5%) all-cause deaths in White AYA SMs compared with 0 out of 89 in Black AYA SMs (log-rank test *P* = 0.0198). Compared with SEER, SMs had lower all-cause mortality (aHR, 0.52; 95% CI, 0.38–0.72) and disease-specific mortality (aHR, 0.29; 95% CI, 0.15–0.59). Post-9/11 deployment did not adversely affect survival among SMs with CHL, highlighting the need for exposure-specific and survivorship-focused research. Survival disparities by race were not appreciated, and mortality in SMs was lower than SEER, highlighting access to care within the Military Health System (MHS) and the healthy soldier effect.

**Significance:**

In this national cohort of 752 U.S. SMs with CHL, post-9/11 deployment and deployment-related variables did not adversely affect overall or disease-specific survival. These findings suggest that deployment exposures or occupational demands do not meaningfully alter CHL outcomes. Lower mortality compared with SEER underscores the healthy soldier effect and highlights the advantages of universal access to timely, coordinated cancer care within the MHS.

## Introduction

Classic Hodgkin lymphoma (CHL) is a relatively rare malignancy in the United States, with approximately 9,000 new cases diagnosed each year (1). With a young median age at diagnosis of 38 years (2), CHL is the most common hematologic malignancy affecting active-duty service members (ADSM; ref. 3). ADSMs represent a uniquely healthy population due to stringent medical, fitness, and behavioral standards, resulting in fewer comorbidities and consistent access to care (4). Prior work has shown that ADSMs with CHL experience superior survival compared with matched U.S. civilians, without racial or rank-based disparities (5), but the impact of deployment on CHL survival is unknown.

Post-9/11 deployments introduced service members (SM) to a wide range of environmental and operational stressors. For example, in recent conflicts in Iraq and Afghanistan, ADSMs faced potential exposure to carcinogenic hazards from burn pits, particulate matter released during blast injuries, and exhaust fumes, among others (6). Although it is unclear if such exposures alter the biology or prognosis of CHL, mortality is a critical endpoint in understanding long-term health outcomes (7). Deployment could plausibly affect survival through trauma-related comorbidities (8–10) and mental health sequelae (11), which could create challenges in delivering curative therapy or meeting eligibility criteria for clinical trials, but the relationship between deployment and survival after CHL has not been evaluated.

To address this gap, we compared survival among ADSMs with CHL who deployed after 9/11 to those who did not deploy after 9/11 and examined whether deployment-specific variables such as location and duration impact mortality. As an exploratory endpoint, we assessed survival by race and compared the survival of ADSMs to civilians from the Surveillance, Epidemiology, and End Results (SEER) database, using the same age range and diagnosis year range.

## Materials and Methods

### Cohort studied

TRICARE beneficiaries with CHL were identified from the U.S. Department of War cancer registry. CHL cases were identified based on the International Classification of Diseases for Oncology histology, third edition, 9650 to 9667, which is the coding system used by the cancer registry. Inclusion criteria were TRICARE beneficiaries who served in the U.S. military and were diagnosed with CHL between 2001 and 2022 while either in active duty or retirement status. Those who had been diagnosed with CHL prior to a post-9/11 deployment, as well as dependents, were excluded from the analysis. Patients with nodular lymphocyte predominant Hodgkin lymphoma were excluded. Because this was a retrospective registry-based study, the cohort sizes were determined by all eligible cases in the Department of War cancer registry during the study period, and therefore, no *a priori* power calculation was performed. Dates of death and causes of death were obtained from the Centers for Disease Control and Prevention National Death Index up through 2022. Causes of death were categorized as disease-specific, secondary cancer, cardiovascular disease, external causes (motor vehicle accidents, injury, accidental poisoning, suicide, and war operations), or other natural causes. The Defense Manpower Data Center (DMDC) was then queried to obtain data on deployment history, including dates of deployment, deployment location, and military occupational specialty codes during deployment. Identified TRICARE beneficiaries meeting inclusion and exclusion criteria were not randomized but rather allocated to two cohorts by their deployment history: those who deployed after 9/11 (9/11 DEP) and those without post-9/11 deployments (NON-9/11 DEP). This designation reflects only the absence of a recorded deployment; routine domestic or overseas duty assignments are not captured in DMDC.

### Statistical analysis

The primary objectives were (i) to compare the survival of the 9/11 DEP cohort with the NON-9/11 DEP cohort and (ii) to assess the impact of deployment variables on survival among those with post-9/11 deployment. Categorical data were summarized using percentages and analyzed using *χ*^2^ statistics. Continuous variables were summarized as means with SDs and compared between the two independent groups using a two-tailed, unpaired Student *t* test, assuming normally distributed underlying populations and equal variances. Survival time was calculated from the date of diagnosis to the date of last follow-up or death. Cox proportional hazards regression model was used to estimate hazard ratios (HR) and 95% confidence intervals (95% CI). Models for all-cause mortality, disease-specific mortality, and mortality by deployment variables were adjusted for potential confounders including age at diagnosis, sex, race, ethnicity, active-duty status, military branch, stage at diagnosis, and year of diagnosis. Among the 9/11-DEP cohort, there were too few patients in occupation subsets to assess the impact on survival. For the exploratory analyses, the log-rank test was used to compare deaths between Black and White ADSMs, recognizing that CHL in younger adults shows age- and race-related differences in EBV-associated disease that support age-stratified analyses (12). The SEER Research Plus Data 17 Registries, November 2024 (13), was used to compare the survival of ADSMs with civilians of the same age range and year of diagnosis range. Because Military Treatment Facilities do not report to SEER and none are located within SEER-17 catchment areas, the likelihood that ADSMs or retirees appear in SEER is negligible, and SEER therefore remains an appropriate comparator for military cancer epidemiologic studies (3, 14). All analyses were conducted using SAS, version 9.4 (SAS, Inc.). This study was determined to be exempt from full Institutional Review Board review (#22-14866). Clinical data used in this study are available from the Joint Pathology Center upon request.

## Results

### Baseline demographics

A total of 752 military personnel with CHL were included, of whom 383 (51%) had not previously deployed after 9/11 (NON-9/11 DEP) and 369 (49%) did deploy after 9/11 (9/11 DEP). Baseline demographics are shown in Table 1. Most military personnel were White males and served in the U.S. Army. The majority were active-duty at the time of their CHL diagnosis, and the nodular sclerosis subtype of CHL was the most common subtype identified. The average age at diagnosis was similar between cohorts (33.5 and 32.1 years among NON-9/11 DEP and 9/11 DEP, respectively, *P* = 0.1151). The NON-9/11 DEP cohort had more military retirees and less active-duty members at the time of CHL diagnosis compared with the 9/11 DEP cohort (36.3% vs. 16.3% and 63.7% vs. 83.7%, respectively, *P* < 0.0001). The 9/11 DEP cohort had more men than the NON-9/11 DEP group (88.3% vs. 80.2%, *P* = 0.0021). There were no differences in CHL stage at diagnosis, subtype, year of diagnosis, race, ethnicity, or branch of service between the 9/11 DEP and NON-9/11 DEP cohorts.

**Table 1.** Baseline demographics of NON-9/11 DEP and 9/11 DEP ADSMs.

VariableLEV	NON-9/11 DEP (*N* = 383)	9/11 DEP (*N* = 369)	*P* value
Age at diagnosis (years)	​	​	​
Mean ± SD	33.5 ± 15	32.1 ± 8.7	0.1151
Median (range)	28 (19–80)	30 (20–66)	0.0059
Sex, *n* (%)	​	​	0.0021
Men	307 (80.2)	326 (88.3)	​
Women	76 (19.8)	43 (11.7)	​
Race, *n* (%)	​	​	0.8375
White	277 (72.3)	260 (70.5)	​
Black	57 (14.9)	60 (16.3)	​
Other	49 (12.8)	49 (13.3)	​
Ethnicity, *n* (%)	​	​	0.6626
Non-Hispanic	297 (77.5)	293 (79.4)	​
Hispanic	22 (5.7)	23 (6.2)	​
Unknown	64 (16.7)	53 (14.4)	​
Active duty, *n* (%)	​	​	<0.0001
Yes	244 (63.7)	309 (83.7)	​
No (retired)	139 (36.3)	60 (16.3)	​
Branch of service, *n* (%)	​	​	0.0560
Army	108 (28.2)	136 (36.9)	​
Air Force	82 (21.4)	76 (20.6)	​
Navy	82 (21.4)	79 (21.4)	​
Marines	46 (12)	35 (9.5)	​
Other/unknown	65 (17)	43 (11.7)	​
CHL subtype, *n* (%)^a^	​	​	0.3475
NSCHL	242 (63.2)	224 (60.7)	​
MCCHL	28 (7.3)	38 (10.3)	​
LRCHL	0	0	​
LDCHL	0	0	​
HLNOS	113 (29.5)	107 (29)	​
Stage at diagnosis, *n* (%)	​	​	0.9239
Stage I	39 (10.2)	33 (8.9)	​
Stage II	158 (41.3)	159 (43.1)	​
Stage III	55 (14.4)	49 (13.3)	​
Stage IV	48 (12.5)	51 (13.8)	​
Unknown	83 (21.7)	77 (20.9)	​
Year of diagnosis, median (range)	2011 (2001–2022)	2012 (2002–2022)	0.1636

a HLNOS, HL not otherwise specified; LDCHL, lymphocyte-depleted CHL; LRCHL, lymphocyte-rich CHL; MCCHL, mixed cellularity CHL; NSCHL, nodular sclerosis CHL.

#### Survival by post-9/11 deployment

**Figure 1.**
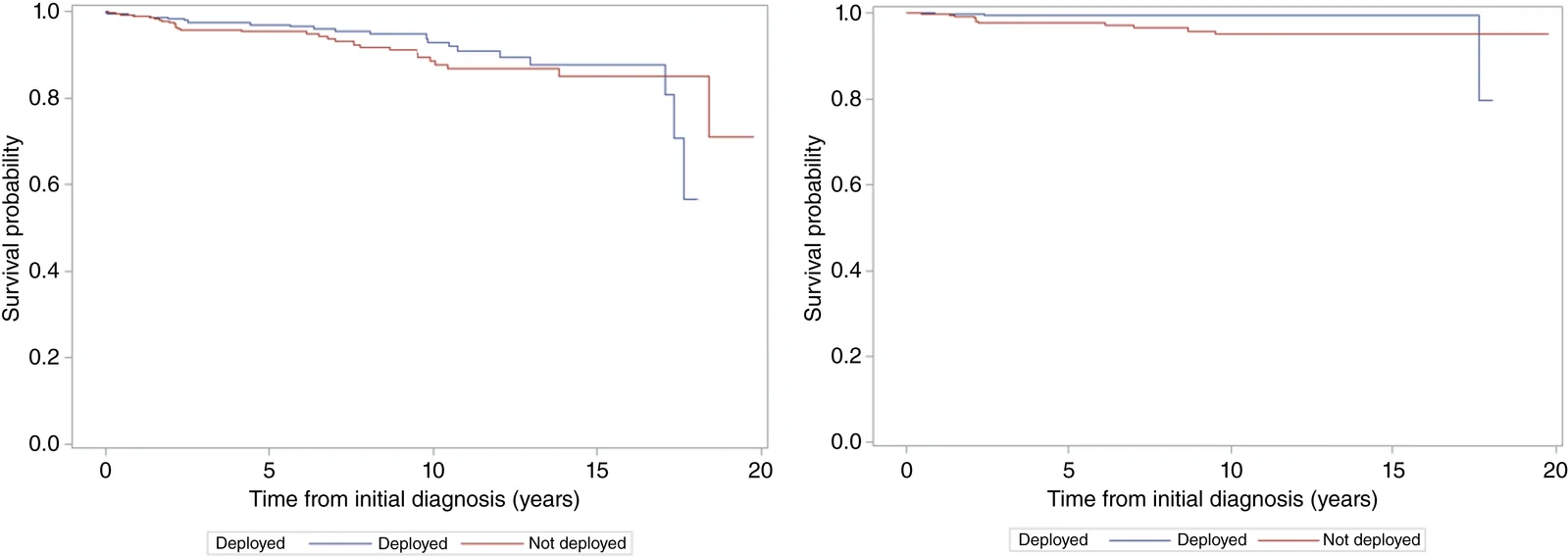
Overall survival (left) and Hodgkin lymphoma–specific survival (right) among military personnel by deployment status.

Causes of death and time to death in both cohorts are shown in Table 2. The median follow-up was 6 years (IQR 2.6–10.5) for the entire cohort, 5.4 years (IQR 2.2–10.7) for NON-9/11 DEP, and 6.5 years (IQR 3.2–10.3) for 9/11 DEP. There were no significant differences in all-cause mortality and disease-specific mortality between the NON-9/11 DEP and 9/11 DEP cohorts, as shown in Fig. 1, even after adjustment for age at diagnosis, sex, race, ethnicity, active-duty versus retiree status, military branch of service, stage, and year of diagnosis. There were 29 total deaths (7.6%) in the NON-9/11 DEP cohort and 23 deaths (6.2%) in the 9/11 DEP cohort [adjusted HR (aHR), 1.64; 95% CI, 0.84–3.19]. The median time to death was 4.2 years (IQR 1.7–8.7) in the NON-9/11 DEP cohort and 6.3 years (IQR 1.9–10.7) in the 9/11 DEP cohort.

**Table 2.** Survival among NON-9/11 DEP and 9/11 DEP ADSMs.

​Mortality	NON-9/11 DEP (*N* = 383)		9/11 DEP (*N* = 369)		HR (95% CI)	aHR (95% CI)^a^
	*n* (%)	Median Time to death (years, IQR)	*n* (%)	Median Time to death (years, IQR)		
All-cause mortality	29 (7.6)	4.2 (1.7, 8.7)	23 (6.2)	6.3 (1.9, 10.7)	0.85 (0.49–1.48)	1.64 (0.84–3.19)
HL-specific mortality	11 (2.9)	2.1 (1.5, 7)	3 (0.8)	2.4 (0.9, 17.6)	0.28 (0.08–1)	0.67 (0.13–3.55)
Other mortality	18 (4.7)	6.6 (1.7, 9.9)	20 (5.4)	6.7 (2.2, 10.6)	1.23 (0.64–2.35)	—
Secondary cancer	9 (2.3)	7.6 (1.9, 10.1)	9 (2.4)	9.8 (7, 12.1)	1.09 (0.43–2.76)	—
CV disease^b^	2 (0.5)	11.3 (4.2, 18.4)	3 (0.8)	10.5 (0, 17.1)	3.40 (0.35–32.94)	—
External causes^c^	2 (0.5)	3.8 (0.8, 6.8)	5 (1.4)	5.6 (4.4, 6.3)	2.39 (0.46–12.35)	—
Other	5 (1.3)	2.3 (1.7, 7.8)	3 (0.8)	2.5 (0.7, 2.5)	0.58 (0.14–2.44)	—

a Model adjusted for age at diagnosis, sex, race, ethnicity, active-duty status, military branch, stage, and year of diagnosis.

b CV, cardiovascular.

c External causes = motor vehicle accident, injury, accidental poisoning, suicide, and war operations.

Disease-specific mortality from CHL occurred in 11 people (2.9%) in the NON-9/11 DEP cohort after a median time of 2.1 years (IQR 1.5–7) and three people (0.8%) in the 9/11 DEP cohort after a median time of 2.4 years (IQR 0.9–17.6; aHR, 0.67; 95% CI, 0.13–3.55). There were nine deaths from secondary cancers in each cohort (2.3% vs. 2.4%; HR, 1.09; 95% CI, 0.43–2.76), occurring after a median time of 7.6 years (IQR 1.9–10.1) in the NON-9/11 DEP cohort and 9.8 years (IQR 7–12.1) in the 9/11 DEP cohort. The most common cause of death from a secondary cancer was non-Hodgkin lymphoma, occurring in three individuals from the NON-9/11 DEP cohort and five individuals from the 9/11 DEP cohort. Other causes of death from secondary cancers included lung cancer in two individuals; esophageal cancer, pancreatic cancer, kidney cancer, and unknown primary in the NON-9/11 DEP cohort; and pancreatic cancer, bone cancer, melanoma, and multiple primary cancers in the 9/11 DEP cohort. Deaths from cardiovascular disease, external causes, and other causes were rare and not statistically different between the NON-9/11 DEP and 9/11 DEP cohorts.

#### Deployment variables and survival

Among the 369 ADSMs in the 9/11 DEP cohort, variables assessed include deployment location to Iraq or Afghanistan, number of deployments, deployment length, and number of locations per deployment, as shown in Table 3. Of the 369 in the 9/11 DEP cohort, 68 (18.4%) deployed to Iraq, 57 (15.5%) deployed to Afghanistan, 32 (8.7%) deployed to both Iraq and Afghanistan, and 212 (57.5%) deployed to another location. There was an even distribution in the number of days deployed: <6 months, 6 months to 1 year, 1 to 2 years, and 2 or more years. Similarly, there was an even distribution in the number of locations deployed to: 1 or 2 locations, 3 to 5 locations, or 6 or more locations. In terms of the number of post-9/11 deployments, 155 deployed once, 81 deployed twice, and 133 deployed 3 or more times. After adjusting for age at diagnosis, sex, race, ethnicity, active-duty versus retiree status at the time of CHL diagnosis, military branch of service, stage, and year of diagnosis, the only deployment variable that affected mortality was 3 to 5 locations per deployment when compared with the reference of 1 to 2 locations per deployment (aHR, 4.29; 95% CI, 1.16–15.84).

**Table 3.** Impact of deployment characteristics on survival in 9/11 DEP ADSMs.

​Variable	9/11 DEP (*N* = 369)		
	*n* (%)	All-cause mortality, *n* (%)	aHR (95% CI)^a^
OIF/OEF location	​	​	​
Iraq	68 (18.4)	7 (10.3)	—
Afghanistan	57 (15.5)	4 (7)	0.59 (0.14–2.52)
Iraq and Afghanistan	32 (8.7)	1 (3.1)	0.22 (0.02–2.06)
Other	212 (57.5)	11 (5.2)	0.41 (0.13–1.30)
Number of deployments	​	​	​
1	155 (42)	11 (7.1)	—
2	81 (22)	3 (3.7)	0.68 (0.17–2.69)
3+	133 (36)	9 (6.8)	1.21 (0.40–3.62)
Deployment length	​	​	​
<6 months	83 (22.5)	5 (6)	—
6 to <12 months	121 (32.8)	5 (4.1)	0.97 (0.25–3.70)
12 to <24 months	99 (26.8)	9 (9.1)	1.84 (0.49–6.93)
24+ months	66 (17.9)	4 (6.1)	1.48 (0.29–7.53)
Locations per deployment	​	​	​
1–2	126 (34.2)	6 (4.8)	—
3–5	106 (28.7)	8 (7.5)	4.29 (1.16–15.84)
6+	137 (37.1)	9 (6.6)	2.67 (0.73–9.78)

a Model adjusted for age at diagnosis, sex, race, ethnicity, active-duty status, military branch, stage, and year of diagnosis.

In terms of latency, 57 (12.5%) of the 9/11 DEP cohort were diagnosed with CHL 0 to 1 year after deployment, 169 (45.8%) were diagnosed 1 to 4 years after deployment, 91 (24.7%) were diagnosed 5 to 10 years after deployment, and 52 (14.1%) were diagnosed 10+ years after deployment.

**Figure 2.**
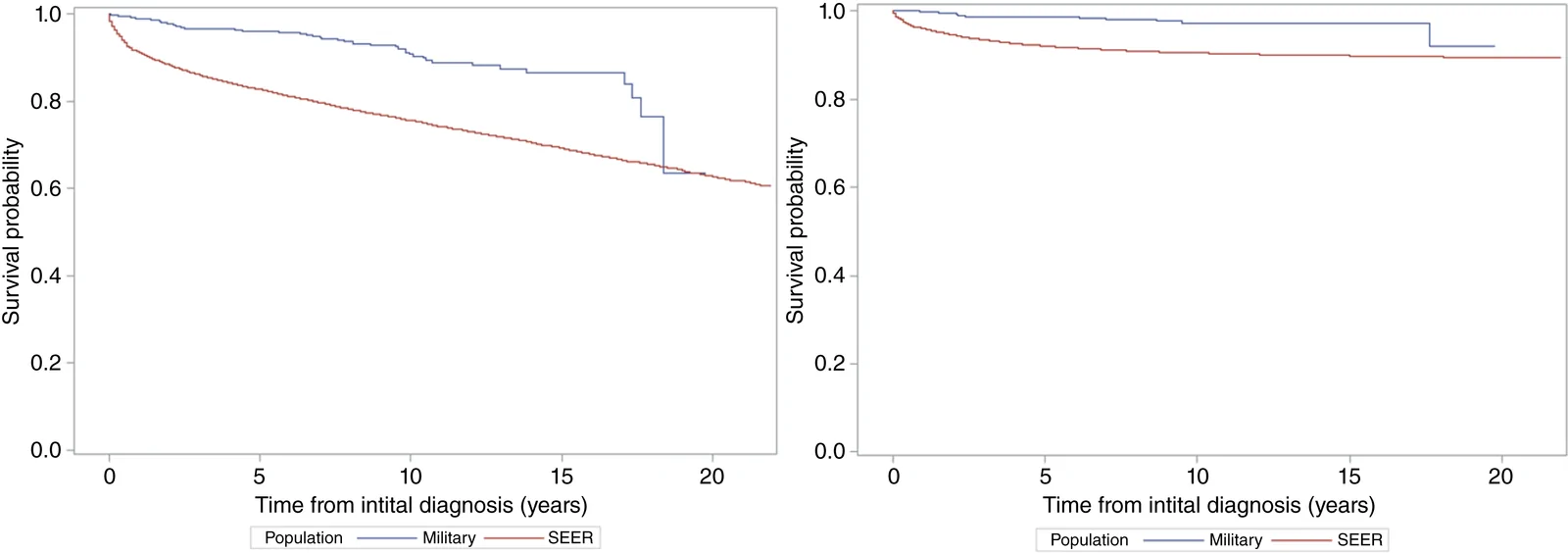
Overall survival (left) and Hodgkin lymphoma–specific survival (right) between military personnel and matched civilians from the SEER database.

Among military personnel diagnosed with CHL below age 40 years, the median follow-up for White and Black ADSMs was 6.1 years (IQR 2.9–10.7) and 7 years (IQR 2.9–11.4), respectively. In terms of deaths, 21/417 (5%) all-cause deaths occurred in White ADSMs versus 0/89 in Black ADSMs (log-rank test *P* = 0.0198). There were no differences in mortality between Black and White ADSMs diagnosed with CHL at or above 40 years of age. When compared with the SEER database, ADSMs with HL had lower all-cause mortality (aHR, 0.52; 95% CI, 0.38–0.72) and disease-specific mortality (aHR, 0.29; 95% CI, 0.15–0.59), as shown in Fig. 2, as well as mortality from other causes (aHR, 0.66; 95% CI, 0.46–0.94), as shown in Table 4.

**Table 4.** Survival among ADSMs compared with SEER.

​Mortality	Military population (*N* = 729)		SEER population (*N* = 40,380)		HR (95% CI)	aHR (95% CI)^a^
	*n* (%)	Median Time to death (years, IQR)	*n* (%)	Median Time to death (years, IQR)		
All-cause mortality	52 (7.1)	5.9 (1.8, 9.8)	9,755 (24.2)	2.3 (0.5, 7.1)	0.32 (0.25–0.42)	0.52 (0.38–0.72)
HL-specific mortality	14 (1.9)	2.2 (1.5, 7)	3,317 (8.2)	1.1 (0.3, 3.2)	0.23 (0.14–0.39)	0.29 (0.15–0.59)
Other mortality	38 (5.2)	6.6 (1.9, 10.1)	6,438 (15.9)	3.9 (0.6, 9.3)	0.37 (0.27–0.51)	0.66 (0.46–0.94)

a Model adjusted for age at diagnosis, sex, race, ethnicity, tumor stage, and year of diagnosis.

## Discussion

In this national cohort of U.S. SMs with CHL, prior post-9/11 deployment was not associated with differences in all-cause or disease-specific mortality, nor mortality from other causes. The loss of significance in the association between deployment and disease-specific survival after adjustment reflects residual healthy deployer effects that are not fully captured by measured covariates. Deployment-related characteristics, including location, duration, and number of deployments, were also not associated with mortality. One intermediate category, deployment to 3 to 5 locations, demonstrated a higher HR; however, this association was not observed in adjacent categories and did not follow a dose–response pattern. Overall, these findings indicate that deployment itself and the operational variability inherent to military service do not seem to influence mortality after CHL within the Military Health System (MHS). Despite CHL being considered a presumptive diagnosis for service-connected toxic exposures (15), the absence of increased disease-specific mortality among previously deployed ADSMs, despite adjusting for critical confounders, suggests that the disease biology is unlikely to be fundamentally changed because of deployment or deployment-related exposures. Routine training and nondeployment duty can also involve occupational exposures (e.g., weapons systems, munitions, gear), which may overlap with deployment-related exposures and could reduce observable differences between cohorts. Additionally, more than half of deployed SMs who developed CHL were diagnosed within 5 years of returning from deployment, reflecting the overlap between deployment age and the usual age at CHL onset and underscoring the importance of maintaining uninterrupted access to care during this period.

Our results extend prior work (16, 17) showing no increased risk of lymphoid malignancies among post-9/11 deployed personnel by demonstrating that survival after CHL diagnosis is similarly unaffected. Although deployed SMs may encounter environmental hazards or blast-related trauma, which could be a surrogate for carcinogenic exposure (6, 18), if these exposures were endured during post-9/11 deployment, they did not translate into worse survival in this cohort. The equal-access structure of the MHS and the high baseline fitness of active-duty personnel who are worldwide deployable may mitigate potential adverse effects of deployment on treatment delivery or comorbidity burden (8–10).

Although secondary cancer mortality did not differ between the NON-9/11 DEP and 9/11 DEP cohorts, the most common cause of death in this category was non-Hodgkin lymphoma. Survivors of CHL are known to have a markedly increased risk of secondary non-Hodgkin lymphoma (19), and certain histologies carry worse outcomes than *de novo* disease (20). This pattern functions as an internal validity check, supporting the accuracy of our dataset.

As an exploratory analysis, we assessed mortality by race and compared mortality between ADSMs and the SEER database. Among those diagnosed with CHL under age 40 years, Black CHL survivors had fewer deaths compared with White CHL survivors, which contrasts with civilian data (21). This difference likely reflects the very small number of deaths in both groups, and the absence of racial disparities in this cohort is consistent with the equal access to care and reduced socioeconomic barriers within the active-duty and retiree populations (5). The finding of lower all-cause and disease-specific mortality among ADSMs compared with the general population from SEER has been described in the literature and reflects the healthy soldier effect as well as comprehensive access to not only oncology care but also primary care, preventive medicine, and mental health care (7).

This study has limitations, including the rarity of CHL in this defined military population, which resulted in a cohort size fixed by available cases. In addition to fixed cases, cancer registry data and DMDC do not contain detailed exposure metrics, trauma data, treatment-level information, or patient reported outcomes such as symptoms or quality of life. Future work incorporating exposure-specific data, trauma registries, treatment intensity, and longitudinal survivorship assessments will be important to refine risk stratification in the postdeployment period.

In summary, neither deployment nor deployment-specific variables were associated with worse survival among SMs with CHL. Taken together, these findings likely reflect the combined influence of equal access to care, reduced socioeconomic barriers, and the healthy soldier effect on improved outcomes within the MHS.
